# Angioleiomyoma: A Rare Cause of Fixed Flexion Contracture of the Elbow

**DOI:** 10.1155/SRCM/2006/93569

**Published:** 2006-08-30

**Authors:** Asterios Dramis, Robert J. Grimer

**Affiliations:** Department of Oncology, Royal Orthopaedic Hospital, Birmingham B31 2AP, UK

## Abstract

We describe an unusual case of a patient presented with a painless fixed flexion contracture of the elbow due to an angioleiomyoma. This benign smooth muscle tumour should be considered in the differential diagnosis of flexion contractures of the elbow.

## INTRODUCTION

Angioleiomyoma is a rare, benign smooth muscle tumour that originates in the tunica media of veins. It can occur anywhere in the body and can be found in the dermis, subcutaneous fat, and fascia. This tumour causes pain in approximately 60% of the patients and occurs most commonly on an extremity, particularly the lower leg.

We present a case of angioleiomyoma of the elbow where the presenting complaint was fixed flexion contracture of the elbow.

### Case report

**Figure 1:**
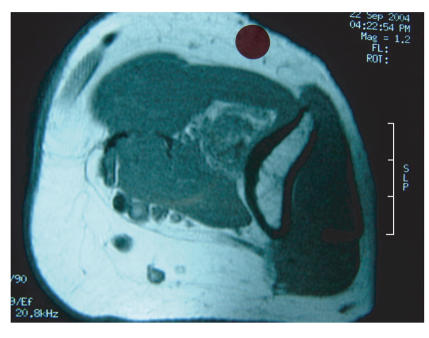
An MRI of the elbow showing a soft tissue tumour.

A healthy 45-year-old woman with no antecedent trauma presented to our department with a 10-year history of problems extending her left elbow. Examination demonstrated 50 degrees of fixed flexion contracture of her left elbow. There were no palpable masses and no convincing tenderness around the elbow. An X-ray and CT scan of the elbow were unremarkable but an MRI scan showed a soft tissue mass lying on the anterior capsule of the elbow, deep to brachialis muscle (Figure 1).

She underwent excision biopsy of the mass. An arm tourniquet was used. Through an anterolateral approach the brachioradialis muscle was identified and reflected laterally. Furthermore the radial nerve was identified and reflected. As brachialis muscle was lifted up a long fatty, vascular mass deep to it was encountered which was about 3 × 1.5 cm in size. It was removed from underlying bone and capsule of elbow joint and sent for histology. A gentle capsular release was performed as well.

An examination under anaesthetic showed that the elbow was now fully extended but was spring due to tight muscles. At the end of the operation, the fixed flexion contracture was 20 degrees. The patient was discharged the following day and outpatient physiotherapy was arranged. At six weeks follow up the patient had full extension of the elbow.

**Figure 2:**
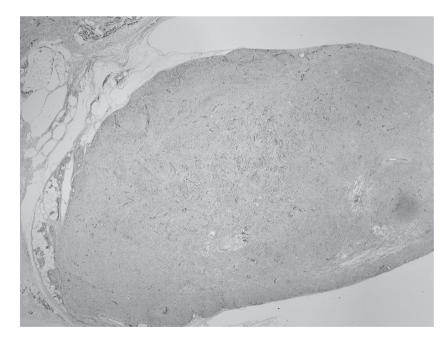
A low magnification histology slide of cavernous-type angioleiomyoma of the patient.

**Figure 3:**
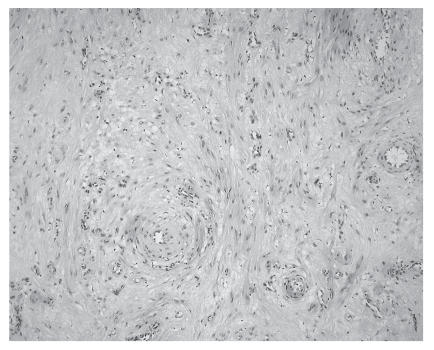
A high-magnification histology slide of the cavernous-type tumour showing dilated vascular channels with little muscular thickening of the walls.

Histological examination showed a tumour, which was composed of dilated vascular channels with smaller amounts of smooth muscle. These findings were consistent with a benign cavernous angioleiomyoma (Figures 2 and 3).

## DISCUSSION

Causes of loss of motion at the elbow are classified as either intrinsic or extrinsic [1] (Table 1).

Our case is unusual because the patient presented with a fixed flexion contracture of the elbow. Therefore, we believe that smooth muscle tumours should be included in the differential diagnosis of fixed flexion contractures of the elbow.

Angioleiomyomas account for 5% of all benign neoplasms of soft tissues [2]. It should be distinguished from all nodular lesions of the extremity like lipomas, ganglion, fibroma, schwannoma, hemangioma, pseudoaneurysm, inclusion cyst, giant cell tumour of tendon sheath, and glomus tumour.

The peak incidence is between the third and sixth decades of life and has a female preponderance.

The most common anatomical site is the lower extremity followed by the upper extremity, the head and trunk [2]. Pain and/or tenderness are the most characteristic subjective complaint in patients with angioleiomyoma [2].

The typical lesion is a solitary, small, slow growing, firm, mobile, subcutaneous nodule, the majority being < 2 cm in size.

The histology shows bundles of mature smooth muscles orientated around blood vessels.

The deep soft tissue tumours, which are often solid, show similar features. In addition, marked degenerative changes with hyalinisation, myxoid changes, and calcification are seen.

Morimoto [3] reviewed 241 cases of angioleiomyoma and classified them into three histological types.

1. Solid: the most common type, which has closely compacted smooth muscle and many small, slit-like vascular channels.
2. Venous: thick, easily identifiable muscular walls distinguish this type.
3. Cavernous: the vascular channels are dilated with less smooth muscles. This is the least common of the three.

Radiologically, a differential diagnosis with intramuscular hemangioma might be considered, as both the latter and the angioleiomyoma tumour share some common features demonstrated on magnetic resonance imaging.

Simple excision biopsy is often curative and morbidity is minimal. There were 25 cases (4.4%) of angioleiomyoma of the elbow in Hachisuga's series, but none presented with painless fixed flexion contracture [4].

In our case the patient presented with a painless, nontrau matic fixed flexion contracture of the elbow, getting worse over 10 years. After excision of the angioleiomyoma, the elbow could be fully extended. It would appear that irritation of the anterior capsule by the tumour probably caused the fixed flexion deformity to develop insidiously.

## CONCLUSION

If no obvious cause for a fixed flexion contracture can be identified, further investigations are justified. In this case, the presence of a benign soft tissue tumour was revealed by the MRI scan, removal of which cured the problem.

## Figures and Tables

**Table 1:** Causes of elbow contractures.

*Extrinsic*	*Intrinsic*

Ectopic bone formation	Cartilage damage
Contractures of capsule	Articular incongruity
Contractures of collateral ligaments	Adhesions

## References

[1] Mansat P, Morrey BF, Hotchkiss RN, Morrey BF (2000). Extrinsic contracture: The column procedure, lateral and medial capsular releases. *The Elbow and Its Disorders*.

[2] Ramesh P, Annapureddy SR, Khan F, Sutaria PD (2004). Angioleiomyoma: a clinical, pathological and radiological review. *International Journal of Clinical Practice*.

[3] Morimoto N (1973). Angiomyoma (vascular leiomyoma): a clinicopathologic study. *Medical Journal of Kagoshima University*.

[4] Hachisuga T, Hashimoto H, Enjoji M (1984). Angioleiomyoma: a clinicopathologic reappraisal of 562 cases. *Cancer*.

